# Supplementary material for: A synthetic metabolic network for physicochemical homeostasis
Source: Nat Commun. 2019 Sep 18;10:4239. doi: 10.1038/s41467-019-12287-2 (PMC6751199; doi:10.1038/s41467-019-12287-2)
Supplement: Supplementary file 1 — Supplementary Information [file 41467_2019_12287_MOESM1_ESM.pdf]

Supplementary Information

A synthetic metabolic network for physicochemical homeostasis

Tjeerd Pols, Hendrik R. Sikkema, Bauke F. Gaastra, Jacopo Frallicciardi, Wojciech M. Śmigiel, Shubham Singh and Bert Poolman

Department of Biochemistry, Groningen Biomolecular Sciences and Biotechnology Institute & Zernike Institute for Advanced Materials, University of Groningen, Nijenborgh 4, 9747 AG Groningen, The Netherlands.

Correspondence to: b.poolman@rug.nl

Supplementary Figures 1-7
Supplementary Tables 1-3
Supplementary References 1-4

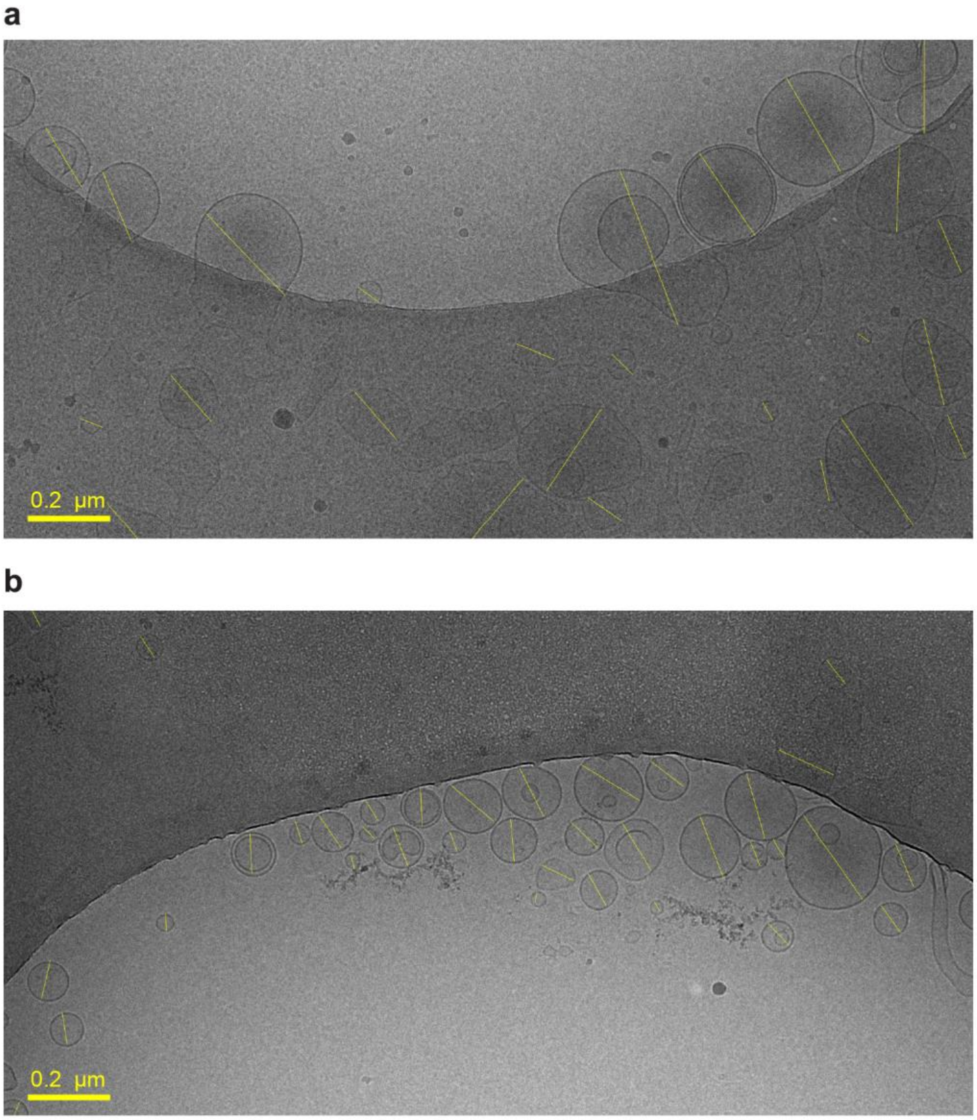

Supplementary Figure 1. Representative CryoTEM images used for size analysis
Representative micrographs showing vesicles extruded through a (**a**) 400 nm polycarbonate filter and (**b**) 200 nm polycarbonate filter; the yellow lines indicate the size measurements. Some of the shapes are somewhat distorted, most likely due to the interaction of the vesicles with the grid.

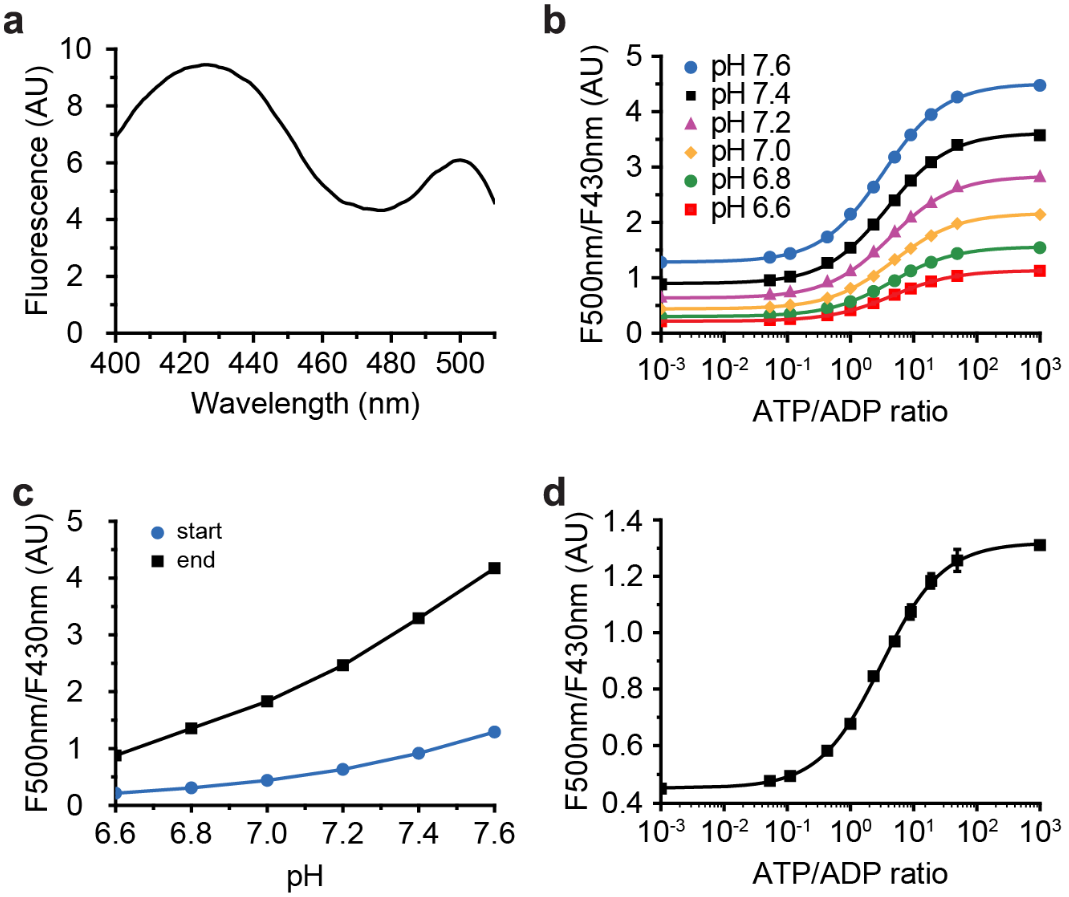

Supplementary Figure 2. Fluorescence-based ATP/ADP ratio sensor PercevalHR. Fluorescence spectrum, calibration and pH dependency of PercevalHR measured in a FP-8300 spectrofluorimeter (Jasco, Inc.). (**a**) Excitation spectrum from 400 nm to 510 nm of PercevalHR encapsulated in the lipid vesicles with an emission wavelength of 550 nm, at equimolar ATP to ADP (2.5 mM each). The spectrum was corrected for background fluorescence. (**b**) Effect of pH on the readout of PercevalHR fluorescence. The ratio of the excitation peaks at 500 nm and 430 nm was measured for different ATP to ADP ratios, measured in 50 mM KPi pH 7.6 (blue circles), pH 7.4 (black squares), pH 7.2 (pink triangles), pH 7.0 (yellow diamonds), pH 6.8 (green circles), and pH 6.6 (red squares), each supplemented with 5.5 mM $MgSO_4$. (**c**) Plot of the start and end values against pH in 50 mM KPi, as fitted with Eq. 3. (**d**) Calibration of PercevalHR inside the lipid vesicles. The ratio of the excitation peaks at 500 nm and 430 nm changes when the ATP to ADP ratio is varied. Error bars indicate the standard deviation of two independent encapsulations. The data points were fit with the Hill equation (black line), as described in the methods (n = 1, k = 3.02, start = 0.46, end = 1.30).

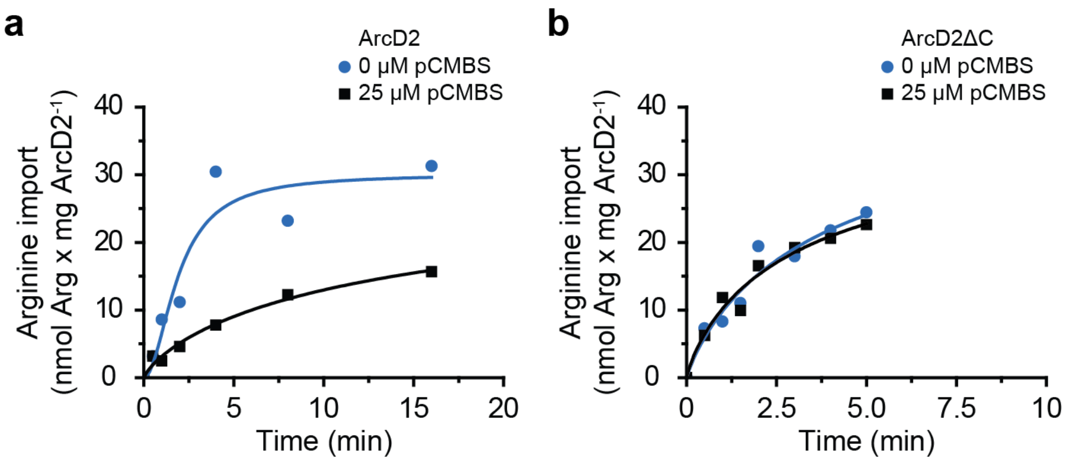

Supplementary Figure 3. pCMBS does not inhibit arginine/ornithine transport by ArcD2ΔC. Radiolabeled arginine uptake by (**a**) wild-type ArcD2 and (**b**) cysteine-less ArcD2 (ArcD2ΔC) in the absence (blue circles) and presence (black squares) of 25 µM of pCMBS. The pCMBS was added 45 minutes prior to the start of the measurement to allow for the binding reaction to occur. The proteoliposomes were loaded with 0.5 mM L-ornithine and the final ^{14}C-L-arginine concentration was 20 µM for the wild-type and 10 µM for the cysteine-less ArcD2. The presented data are obtained from a single experiment, but similar measurements (optimization of assay and labeling) were done multiple times.

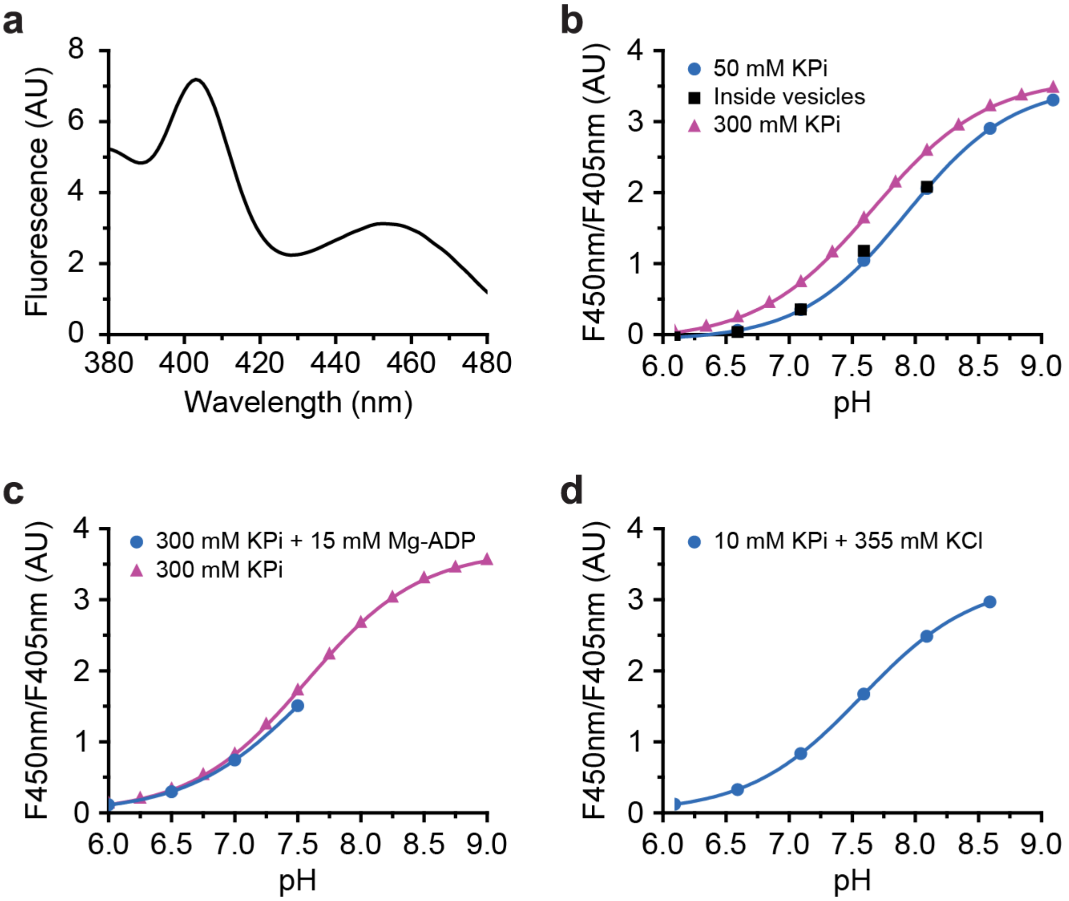

Supplementary Figure 4. Calibration of pyranine inside lipid vesicles. (**a**) Excitation spectrum from 380 nm to 480 nm of pyranine inside the lipid vesicles with an emission wavelength of 512 nm, at pH 7.0. (**b**) Pyranine was measured at varying pH in 50 mM KPi (blue circles), inside the lipid vesicles containing 50 mM KPi (black squares) and in 300 mM KPi (pink triangles), using a FP-8300 spectrofluorimeter (Jasco, Inc.). The ratio of the excitation peaks at 450 nm and 405 nm changes as a function of pH. The data points in 50 and 300 mM KPi were fit with a logistic function (blue and pink traces), as described in the methods (50 mM KPi: L = 3.60, k = 2.38, x_0 = 7.84; 300 mM KPi: L = 3.70, k = 2.21, x_0 = 7.57). The data points inside the unshocked lipid vesicles perfectly match those in 50 mM KPi. (**c**) Similar to panel B, pyranine was measured at varying pH in 300 mM KPi with 15 mM Mg-ADP (blue circles) and 300 mM KPi (pink triangles). Data points in 300 mM KPi plus 15 mM Mg-ADP were fit (blue trace) to obtain: L = 3.22, k = 2.16 and x_0 = 7.56. (**d**) Similar to panel B, pyranine was measured at varying pH in 10 mM KPi plus 355 mM KCl (blue circles). Data points in 10 mM KPi plus 355 mM KCl were fit (blue trace) to obtain: L = 3.27, k = 2.23 and x_0 = 7.48.

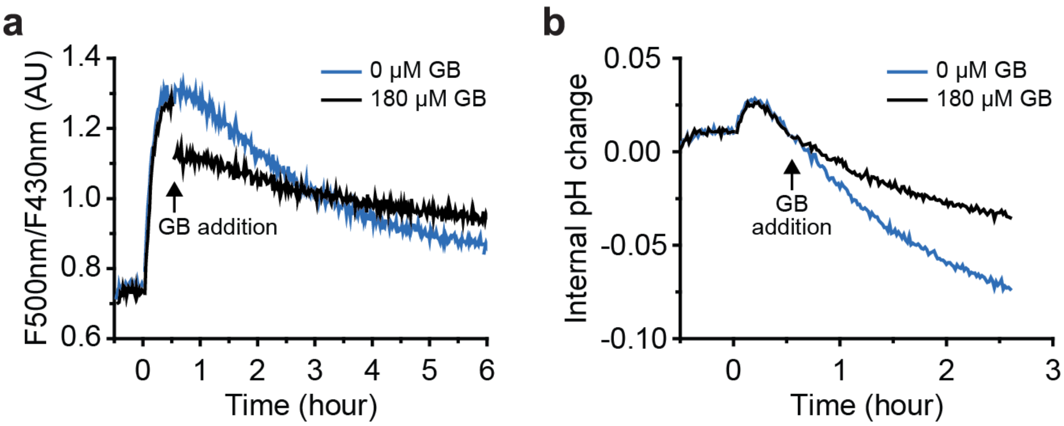

Supplementary Figure 5. ATP production and pH changes in arginine-metabolizing vesicles obtained by extrusion through 200 nm polycarbonate filters. (**a**) Analogous to Fig. 6a, the effect of glycine betaine (GB) import on ATP production as measured by PercevalHR fluorescence (protocol B3) in arginine-metabolizing vesicles exposed to an osmotic upshift (addition of 250 mM KCl) in the presence (black trace) and absence (blue trace) of 180 µM glycine betaine (added at t = 0.5 h); 5 mM arginine was added at t = 0 (*n*=2). (**b**) Analogous to Fig. 6d, the effect of glycine betaine on the internal pH measured by pyranine (protocol B4) in arginine-metabolizing vesicles exposed to an osmotic upshift (250 mM KCl) in the presence (black trace) and absence (blue trace) of 180 µM glycine betaine (added at t = 0.5 h); 5 mM arginine was added at t = 0 (*n*=2).

a

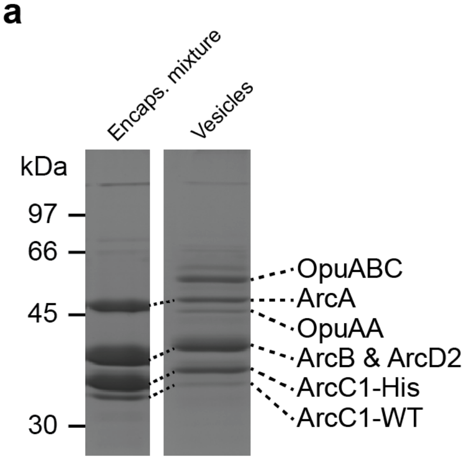

b

	Encaps. mixture	Inside vesicles
ArcA	1	1
ArcB	1.8	1.9
ArcC1	1.6	1.4

Supplementary Figure 6. SDS-Polyacrylamide gel electrophoresis of purified and encapsulated proteins. (**a**) On the left, encapsulation mixture containing 0.94 µg of ArcA, 2.4 µg of ArcB and 1.8 µg of ArcC1. On the right, the vesicles with encapsulated proteins and co-reconstituted ArcD2 and OpuA. OpuA consists of two separate proteins: OpuABC, containing the transmembrane domain fused to the substrate-binding domain; and OpuAA, the nucleotide-binding domain fused to a regulatory domain. Although ArcB and ArcD2 have a different monomeric molecular weight (40.9 and 56.7 kDa respectively), ArcD2 migrates at a similar position as ArcB, which has been observed for many other membrane proteins[1]. The purification of his-tagged ArcC1 (ArcC1-His) always yields a small amount of wild type ArcC1 (ArcC1-WT), because the cell expresses ArcC1-WT at a basal level and heterooligomers are formed; the two proteins do migrate at a different position. (**b**) Quantification of the ratio of the ArcA, ArcB and ArcC1 before (encapsulation mixture) and after reconstitution (inside the vesicles) as analyzed with ImageJ (standard deviation from analyzing 4 independent loadings is 0.1). The ratio inside the vesicles does not differ significantly from that of the encapsulation mixture.

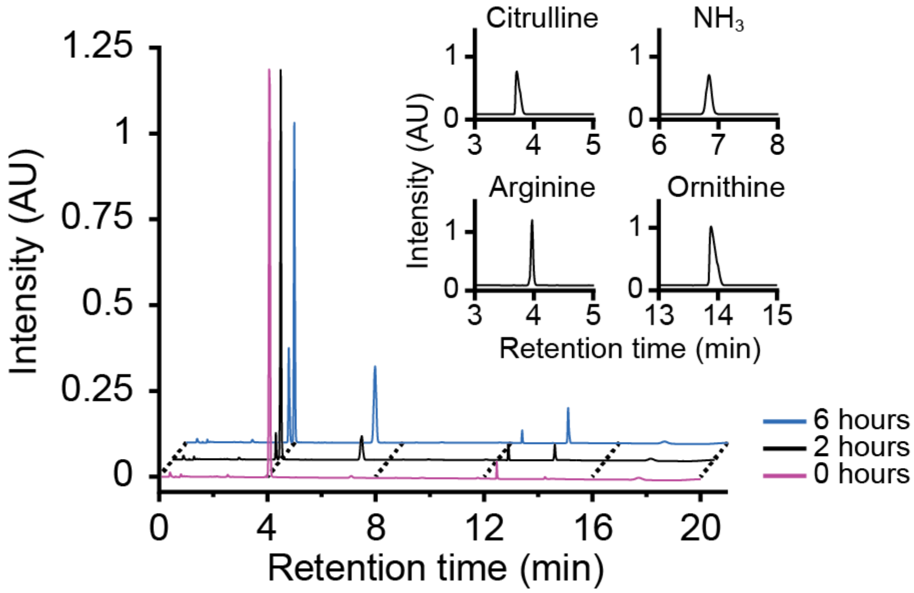

Supplementary Figure 7. Raw spectra from HPLC chromatograms. Representative chromatograms of amino acids and ammonia produced by the vesicles containing the arginine breakdown pathway. Chromatograms were recorded at 269 nm. As expected, at t = 0 only 5 mM arginine is detected (at retention time = 4 min). Citrulline, NH_3 and ornithine (at retention time 3.8; 6.8 and 14 min, respectively) appear at the expense of arginine in the chromatograms of samples taken at 2 and 6 hours. Inset: Truncated chromatograms of standard solutions containing 5 mM of a single amino acid or NH_3. Such chromatograms were used to determine the retention time and to calibrate the signals.

Supplementary Table 1. Primers used for cloning

Primer	Sequence (5’ -> 3’)
arcA-Fw	AAACCATGGGTATGAACAATGGAATTAAATGTTAACTCAGAAATTGGG
arcA-Rv	ATAAGGATCCCAAATCTTCACGCCAAAGTGGTTGTGAC
arcB-Fw	AAACCATGGGTATGACATCACCACTTATTACAAAAGCAGAAGTAAAC
arcB-Rv	ATAAGGATCCTTTAAAATCTTCAGGAACTGCTGGGATAAATAAATTAC
arcC1-Fw	ATGGTGAGAATTTATATTTTCAAGGTGTAAAACGTATTGTTGTAGCCC
arcC1-Rv	TGGGAGGGTGGGATTTTCATTAAGGAACAATTCTTGTTCCACTAC
arcD2-Fw	ATGGGTGGTGGATTTGCTATGGAAAACAAGAAAACAAAAGGG
arcD2-Rv	TTGGAAGTATAAATTTTCGTAGCCTAGTAATCCCCG
*arcD2*ΔC-1	AGCTGTAATUATGATTACTTATGCTTTGGTTGGAGC
*arcD2*ΔC-2	AGACCAGAAUTGCTAAAACTCCTAGTAAGG
*arcD2*ΔC-3	ATTCTGGTCUTGATTACTGGCGCAAAATCAGGAAC
*arcD2*ΔC-4	AATTACAGCUGTTGCCATATAAATAAAGAC

Supplementary Table 2. Strains used in this study

Strain	Genotype	Vector	Reference
L. lactis NZ9000<br>*L. lactis* NZ9000-A<br>*L. lactis* NZ9000-B<br>*L. lactis* NZ9000-C1	MG1363 with *nisRK* in *pepN* locus	 pNZ*arcA*<br>pNZ*arcB*<br>pNZ*arcC1*	2<br>This study<br>This study<br>This study
L. lactis JP9000<br>*L. lactis* JP9000-D2<br>*L. lactis* JP9000-2ΔC	MG1363 with *nisRK* in pseudo_10 locus	 pNZ*arcD2*<br>pNZ*arcD2*ΔC	3<br>This study<br>This study
E. coli BL21(DE3)	F^- *ompT gal dcm lon* $hsdS_B(r_B^-\ m_B^-)$ λ(DE3 [*lacI lacUV5-T7 ind1 sam7 nin5*])	pRsetB-PercevalHR	This study
L. lactis OPU401	NZ9000 Δ*opuA*	pNZ*opuA*His	4

Supplementary Table 3. Enzyme activities for data presented in Fig. 6b

Buffer	ArcA	ArcB	ArcC1
50 mM KPi	2.9 ± 0.3	233.3 ± 52.3	362.9 ± 22.8
300 mM KPi	4.8 ± 2.9	128.9 ± 49.7	135.0 ± 76.8

All values are given in µmol * min^{-1} * mg^{-1}. Errors indicate standard deviation.

Supplementary References

1. Rath, A., Glibowicka, M., Nadeau, V. G., Chen, G., Deber, C. M. Detergent binding explains anomalous SDS-PAGE migration of membrane proteins. *Proc. Natl. Acad. Sci. U.S.A.* **106,** 1760–1765 (2009).
2. Kuipers, O. P., de Ruyter, P. G. G. A., Kleerebezem, M., de Vos, W. M. Quorum sensing-controlled gene expression in lactic acid bacteria. *J. Biotechnol.* **64.1,** 15-21 (1998).
3. Noens, E. E. E., Kaczmarek, M. B., Zygo, M., Lolkema, J. S. ArcD1 and ArcD2 arginine/ornithine exchangers encoded in the arginine deiminase pathway gene cluster of *Lactococcus lactis*. *J. Bacteriol.* **197.22,** 3545-3553 (2015).
4. Geertsma, E. R., Nik Mahmood, N. A. B., Schuurman-Wolters, G. K., Poolman, B. Membrane reconstitution of ABC transporters and assays of translocator function. *Nat. Protoc.* **3,** 256–266 (2008).
